# Hippocampal Volume Reduction in Congenital Central Hypoventilation Syndrome

**DOI:** 10.1371/journal.pone.0006436

**Published:** 2009-07-30

**Authors:** Paul M. Macey, Christopher A. Richard, Rajesh Kumar, Mary A. Woo, Jennifer A. Ogren, Christina Avedissian, Paul M. Thompson, Ronald M. Harper

**Affiliations:** 1 School of Nursing, University of California Los Angeles, Los Angeles, California, United States of America; 2 Brain Research Institute, University of California Los Angeles, Los Angeles, California, United States of America; 3 Department of Neurobiology, University of California Los Angeles, Los Angeles, California, United States of America; 4 Laboratory of Neuro Imaging, Department of Neurology, David Geffen School of Medicine at UCLA, University of California Los Angeles, Los Angeles, California, United States of America; 5 Sleep Disorders Center, St. Joseph Hospital, Orange, California, United States of America; The Research Center of Neurobiology - Neurophysiology of Marseille, France

## Abstract

Children with congenital central hypoventilation syndrome (CCHS), a genetic disorder characterized by diminished drive to breathe during sleep and impaired CO_2_ sensitivity, show brain structural and functional changes on magnetic resonance imaging (MRI) scans, with impaired responses in specific hippocampal regions, suggesting localized injury.

We assessed total volume and regional variation in hippocampal surface morphology to identify areas affected in the syndrome. We studied 18 CCHS (mean age±std: 15.1±2.2 years; 8 female) and 32 healthy control (age 15.2±2.4 years; 14 female) children, and traced hippocampi on 1 mm^3^ resolution T1-weighted scans, collected with a 3.0 Tesla MRI scanner. Regional hippocampal volume variations, adjusted for cranial volume, were compared between groups based on t-tests of surface distances to the structure midline, with correction for multiple comparisons. Significant tissue losses emerged in CCHS patients on the left side, with a trend for loss on the right; however, most areas affected on the left also showed equivalent right-sided volume reductions. Reduced regional volumes appeared in the left rostral hippocampus, bilateral areas in mid and mid-to-caudal regions, and a dorsal-caudal region, adjacent to the fimbria.

The volume losses may result from hypoxic exposure following hypoventilation during sleep-disordered breathing, or from developmental or vascular consequences of genetic mutations in the syndrome. The sites of change overlap regions of abnormal functional responses to respiratory and autonomic challenges. Affected hippocampal areas have roles associated with memory, mood, and indirectly, autonomic regulation; impairments in these behavioral and physiological functions appear in CCHS.

## Introduction

Children with congenital central hypoventilation syndrome (CCHS), a condition marked by a loss of breathing drive during sleep and impaired central sensitivity to CO_2_, show impaired blood pressure and hypoxia responses, unique changes in affect, and problems with memory [1], [2], [3], [4], [5], [6]. The constellation of physiological, memory and affect deficiencies suggests injury to limbic structures which regulate those behaviors.

The hippocampus and its immediate projections serve memory and spatial orientation roles, are implicated in mood regulation, and participate in triggering of breathing onset after a respiratory pause. Structures receiving projections from the hippocampal formation, such as the mammillary bodies, are affected in CCHS [7], as are anterior thalamic nuclei which form part of the hippocampus-fornix-mammillary body-thalamic circuitry participating in memory formation [8], [9], and the projecting fibers from the hippocampus in the fornix show reduced cross-sectional area [7]. Severe volume reduction of the mammillary bodies, such as occurs in patients with chronic alcoholism and Wernicke-Korsakoff's syndrome, is accompanied by significant short-term memory deficits [10]. Memory deficits in CCHS patients are less severe than in Wernicke-Korsakoff's syndrome [5], but the combination of reduced mammillary body volume and functional impairments in behaviors that the structures serve raises the possibility of regional injury to hippocampal structures that project to the mammillary bodies.

The hippocampus and adjacent brain areas show gross deficits in CCHS, as indicated by functional MRI (fMRI) responses to blood pressure [11], [12], CO_2_, and hypoxic challenges [13], [14], and by overall structural changes demonstrated by quantitative MRI techniques [15], [16]. However, the fMRI and structural evaluations have spatial resolution too limited to adequately identify the precise regions affected. Defining the areas within the hippocampus that are injured or developmentally altered in CCHS could improve understanding of the deficits in the syndrome. Working memory, in particular, is more affected in CCHS than other behavioral or cognitive functions [5], and the hippocampus is essential for that aspect of memory [17]. Such memory deficits may stem from altered hippocampal-mammillary body interactions; thus, it is important to determine whether neurons in regions responsible for originating the fornix fibers to the mammillary bodies underlie the injury to the latter structures, and whether other areas within the hippocampus contribute to damage in recipient neurons. Mutations in PHOX2B, a transcription factor instrumental in autonomic ganglia development, are found in over 90% of CCHS children, and are believed to underlie the impaired autonomic and respiratory characteristics of the syndrome [18]. The hippocampus may also be affected by developmental consequences of PHOX2B mutations, since Phox2b expression in the mouse is found in pyramidal and granule cell layers of that structure [19].

The objective of this study was to evaluate hippocampal morphology in CCHS and control children to determine specific sites injured in the syndrome. We hypothesized that CCHS subjects would show tissue loss in the hippocampus within regions that give rise to the fibers of the fornix.

## Methods

### Subjects

Children with CCHS were recruited through the CCHS family network (http://www.cchsnetwork.org) from throughout the United States and Canada, and were all diagnosed according to American Thoracic Society criteria [1]; we included only those CCHS subjects who did not require ventilatory support while awake. All subjects were diagnosed early in life, but we had only caregiver recollections of the age of diagnosis, with many caregivers indicating periods of symptoms extending over months before the syndrome was identified. A subset of six subjects tested positive for the PHOX2B mutation characteristic of the syndrome [18], and two were inconclusive; the other subjects had not been evaluated. Exclusion criteria included other conditions with potentially confounding influences on neural injury, such as cardiovascular and neurological disorders, and diagnosed Hirschsprung's disease. Control subjects were recruited through advertisements at the university campus, and were in good health, without any known problems that could affect brain tissue. All CCHS and control subjects were awake during the entire study (without anesthesia or sedation).

### Ethics

The study protocol was approved by the Institutional Review Board of the University of California at Los Angeles, and all subjects and their parents/guardians gave written consent/assent prior to the study.

### Magnetic Resonance Imaging

Studies were performed in a 3.0 Tesla MRI scanner (Siemens Magnetom Trio, Erlangen, Germany), using a whole-body transmitter coil and a receive-only 8-channel phased-array head-coil. Subjects lay supine, and foam pads were placed on both sides of the head to minimize head motion. Two high-resolution T1-weighted image volumes were collected using a magnetization-prepared-rapid-acquisition-gradient-echo pulse sequence (repetition-time = 2200 ms; echo-time = 3.05 ms; inversion-time = 1100 ms; flip-angle = 10°; matrix size = 256×256; field of view = 220×220 mm; slice thickness = 1.0 mm; 176 slices). At the time of scanning, the anatomical images were visually inspected for motion artifact. If artifacts were seen, the scan was repeated. Proton-density and T2-weighted images were also collected, using a dual-echo turbo spin-echo pulse sequence (repetition-time = 8000 ms; echo-time 1, 2 = 17, 133 ms; flip-angle = 150°; matrix size = 256×256; field of view = 240×240 mm; slice thickness = 5.0 mm; turbo factor = 5), for anatomical evaluation of visible pathology.

A small number of the CCHS subjects (three) were scanned in an alternate 3.0 Tesla scanner (Siemens Allegra, Erlangen, Germany), as the Trio was not available (differences in T1-weighted scanning parameters: repetition-time = 1970 ms; echo-time = 4.38 ms; field of view = 240×240 mm; slice thickness = 1.2 mm; 176 slices). Despite the different scanning protocols, we elected to include these subjects in the final study due to the rare nature of the syndrome, and the restriction on re-scanning of these subjects due to the subsequent implantation of phrenic pacemakers and other devices. The image detail and tissue contrast across the two scanners were deemed similar by the tracers.

### Data Analysis

The anatomical T1-weighted, T2- and PD-weighted images were evaluated to verify the absence of gross brain pathology, such as cystic or other mass lesions.

#### Preprocessing of the Anatomical Images

The two T1-weighted anatomical volumes were combined and processed prior to tracing. The statistical parametric mapping package SPM5 (Wellcome Department of Cognitive Neurology, UK; http://www.fil.ion.ucl.ac.uk/spm/), MRIcroN [20], and Matlab-based (The MathWorks Inc, Natick, MA) custom software were used. The two T1-weighted volumes were realigned and averaged to increase signal-to-noise ratio. Each averaged image volume was shifted and rotated to overlap a standard template (Montreal Neurological Institute, included with SPM5), using a 6-parameter rigid-body (non-distorting) affine transformation. The images were resliced at a 1×1×1 mm resolution, within a standard rectangular volume surrounding the template (i.e., bounding box).

An intensity correction for variations in signal intensity due to field inhomogeneities was performed using the SPM5 unified segmentation [21]. This procedure also segmented gray matter, white matter and cerebrospinal fluid (CSF), producing probability maps for each of these components. These maps were used to calculate total intracranial volume (TIV): for each voxel, the three probabilities were summed and classified as intracranial if the probability was greater than 0.5.

#### Tracing of the Hippocampus

An investigator, blinded to group assignment, traced the left and right hippocampi of each subject. The delineation of the hippocampus was performed according to a standard protocol [22], using MRIcroN software. However, after tracing was completed across the coronal sections as in the standard protocol, the sagittal sections were viewed to ensure consistency along the anterior-posterior direction. In particular, the sagittal view was used to confirm the location of the anterior tip of the hippocampus. A second tracer, also blinded to group assignment and the earlier investigator's results, repeated the tracings on a subset of images (4 CCHS, 4 control) to establish inter-tracer reliability, calculated as sensitivity and specificity of one tracer relative to another in terms of voxels classified as hippocampus, as well as Cronbach's Alpha for global volumes.

#### Three-Dimensional Statistical Analysis

Hippocampal morphometry, the analysis of hippocampal shape, was performed using an established procedure [23]. Differences in brain size were partitioned by scaling the hippocampal tracing based on the ratio of the TIV for each subject to the mean TIV across all subjects; the same scale factor (the cube root of the ratio) was applied to the x, y, and z directions. Each hippocampal trace was fitted with a parametric surface mesh consisting of a fixed number of points. The “surface volume” was defined as the distance from the surface to the medial line, i.e., the anterior-posterior center line of the hippocampus, and was calculated for each point on the mesh; this distance is therefore an estimate of the volume below the surface point. The surface mesh was spatially normalized to a hippocampal template. The process was repeated for all subjects, resulting in equivalent surface points across the hippocampal surface for all subjects. The subjects' surface volumes were compared at each point on the mesh using t-tests, resulting in “maps” across the surface of CCHS and control group differences. Control for multiple comparisons was performed using non-parametric permutation tests [24], which are more robust and are based on fewer assumptions than traditional parametric methods (e.g., Bonferroni correction). (A complete description of permutation testing is available elsewhere [25].) Effect size maps of Pearson's r values at each point were also calculated across the surface.

## Results

### Subjects

We studied 18 CCHS and 32 control subjects, with the two groups having similar age and sex distributions (Table 1). The mean global volumes of brain compartments were the same in both groups for gray matter, but showed modest, non-significant differences in white matter and CSF, with slightly less white matter and more CSF in CCHS. However, when taken as a ratio of brain (gray+white matter) to CSF volume, a significant difference developed, with the CCHS subjects showing lower ratios (Table 1). The brain-to-CSF ratio avoids variability due to brain size differences. The lower brain-to-CSF value in CCHS is consistent with anecdotal visual impressions of patients' scans appearing to show more CSF. However, no major brain pathologies were visible on the T1- or T2-weighted scans (consistent with the definition of CCHS [1]).

**Table 1:** Subject characteristics. GM = gray matter; WM = white matter; CSF = cerebrospinal fluid; TIV = total intracranial volume.

	CCHS		Control		Group Difference(*p*)
N	18		32		
Sex (female:male)	8:10 ♀:♂		14:18♀:♂		*0.96*
	Mean†	Std†	Mean†	Std†	
Age (years)	**15.1**	±2.2	**15.1**	±2.4	*0.9*
GM Volume (liters)	**0.83**	±0.10	**0.83**	±0.11	*0.8*
WM Volume (liters)	**0.44**	±0.07	**0.46**	±0.07	*0.3*
CSF (liters)	**0.14**	±0.04	**0.11**	±0.051	*0.07*
TIV (liters)	**1.45**	±0.19	**1.44**	±0.15	*0.9*
Brain-to-CSF Ratio	**9.8**	±3.2	**12.8**	±4.0	***0.009***
Left hippocampus (mm^3^)	**2139**	±288	**2172**	±297	*0.7*
Right hippocampus (mm^3^)	**2296**	±316	**2280**	±326	*0.9*

Group comparisons for male/female were performed with Chi-square tests, and for other continuous variables were performed with ANCOVA, with all other variables included as covariates.

†Means and standard deviations are adjusted values (by ANCOVA). Hippocampal volumes are unscaled (i.e., not adjusted for head size).

### Shape Analysis and Regional Volume Differences

**Figure 1:**
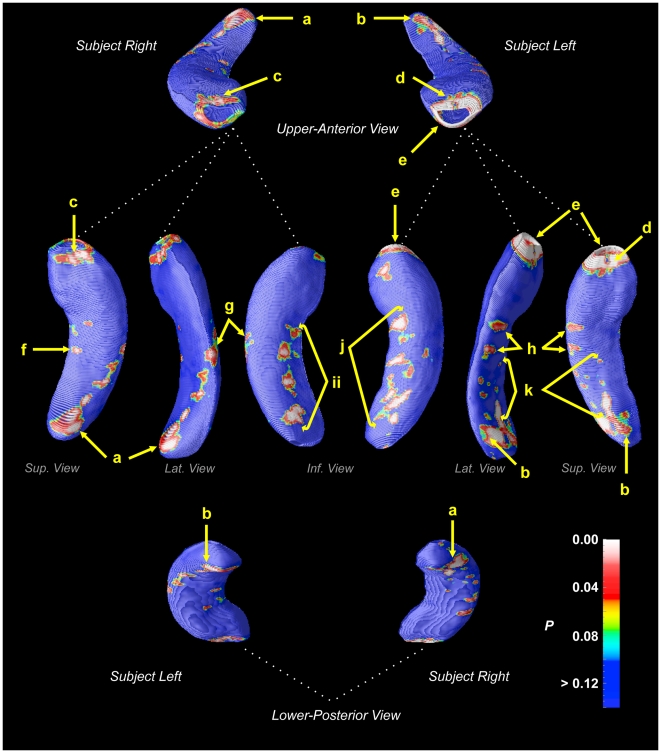
Regions of significant hippocampal volume reduction in CCHS subjects relative to control subjects, color-coded according to significance level (scale bottom-right). Regions: *a, b* – CA1/CA2, near fimbria; *c* right rostral = CA1-CA3, dentate gyrus (DG); *d* left rostral = CA1-CA3, DG; *e* left ventral = CA1, subiculum; *f* = right subiculum; *g, h* = CA1; *i, j* = subiculum; *k* = CA1/CA2.

**Figure 2:**
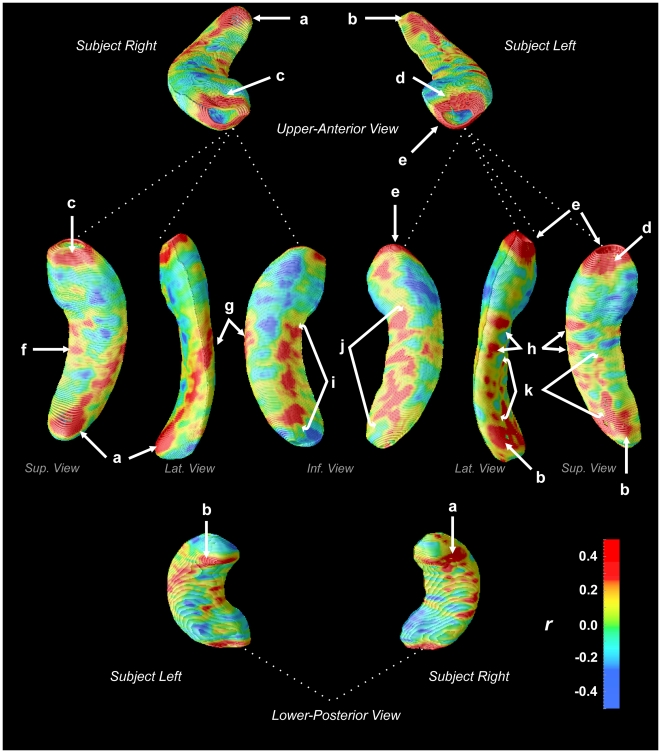
Effect size of hippocampal volume reduction in CCHS subjects relative to control subjects (Pearson's r, scale bottom-right). Regions of positive effect size (yellow-red colors) indicate volume reduction in CCHS. Region labels are the same as Fig. 1: *a, b* – CA1/CA2, near fimbria; *c* right rostral = CA1-CA3, DG; *d* left rostral = CA1-CA3, DG; *e* left ventral = CA1, subiculum; *f* = right subiculum; *g, h* = CA1; *i, j* = subiculum; *k* = CA1/CA2.

Hippocampal morphometry revealed numerous regions of reduced volume in CCHS relative to control subjects (Fig. 1). After controlling for multiple comparisons using permutation testing, a significant overall effect was found in the left hippocampus (*p* = 0.01), but not the right (*p* = 0.1). The regions of significant difference showed moderate to large effect sizes (Fig. 2), with similar magnitudes on both sides; the lower significance on the right side implies greater variability. A number of areas showed no change (green regions in Fig. 2), with a few regions of CA1 and subiculum showing modest (and non-significant) negative effect sizes (blue regions, Fig. 2).

Specific sub-regions, or fields, affected in the left hippocampus (Fig. 1) included the anterior portion, which showed the greatest extent of volume reduction. The left anterior hippocampus showed extensive damage in the most-rostral region, but bilateral changes occurred principally in mid-dorsal areas, corresponding with changes in the lateral regions (CA1/CA2), and on the right mid-section in subiculum.

Inter-tracer reliability for global volumes was 0.87 (Cronbach's alpha); for voxel classifications, sensitivity was 83% and specificity was 93% (second tracer relative to first tracer).

## Discussion

The hippocampus showed localized volume reductions in CCHS subjects, principally on the left side, with many regions affected bilaterally. The changes included reduced volume in bilateral posterior portions of the fimbria, the fibers that form the crus of the fornix, and isolated bilateral regions in CA1 and CA2 from the posterior to mid portions of the structure. Other notable changes appeared in the left extreme-rostral hippocampus. The structural changes are presumably accompanied by impaired function, which should be reflected in behaviors served by the affected areas. Functions associated with the hippocampus include recent and spatial memory, mood, participation in responses to ventilatory and autonomic stimuli [11], [12], [13], [14], [26], and initiation of breathing after central apnea [27]. All of these physiologic, cognitive and mood functions are affected in CCHS children, suggesting that the structural injury shown here contributes to altered functions in the syndrome.

### Other Structural Findings

The reduced hippocampal volumes, and the particular sites of injury may have contributed to the reduced mammillary body volumes and fornix cross sectional areas found in an overlapping patient group [7]. A portion of the output fibers of the hippocampus, including those from the subiculum, comprise the fimbria, affected here, and form the fibers of the fornix, a substantial portion of which project to the mammillary bodies. The hippocampal formation, together with the mammillary bodies and anterior thalamus, serves roles for anterograde and spatial memory, the latter assisted by head direction cells located in the subiculum, lateral mammillary bodies and anterior thalamus [28], [29]. The combined mammillary body and hippocampal changes suggest that compromised spatial memory and orientation are likely present in CCHS. A working memory task that includes a spatial component is affected in some, although not all CCHS subjects [5], and a subset of CCHS subjects show difficulties with spatial concepts [4]. Further spatial processing issues may arise from impaired communication between the hippocampus, mammillary bodies and areas within the tegmentum [8]; the sites within the tegmentum project to vestibular and cerebellar structures [30], [31]. No direct evidence of impaired spatial-motor coordination is available for CCHS, but over half of CCHS children receive physical therapy [4].

The findings of regional hippocampal structural alterations by structure-specific shape analysis procedures are consistent with previous evidence of damage found with whole-brain assessment in CCHS with T2 relaxometry and diffusion tensor imaging techniques [15], [32]. The low resolution of T2 relaxometry and mean diffusivity analyses, combined with the nature of the whole-brain analysis, precluded precise regional examination of the hippocampus in these earlier studies. The T2 technique is also sensitive to different pathologies, as the measure reflects increased water content, but not volume changes. Volume changes, as shown here in the hippocampus, likely result from long-term exposure to injurious processes and possibly developmental differences, whereas increased water content in the tissue could include both longer-term damage as well as acute effects. The morphology findings here confirm the presence of diminished hippocampal tissue, and localize the changes to specific hippocampal regions.

### Brain Functional Changes

The changes in hippocampal structure are reflected in abnormal neural functional responses to physiologic challenges. Hypoxia in CCHS subjects elicits abnormal responses in the right mid-hippocampus [26], while hyperoxia results in aberrant responses in the left posterior hippocampus [13]; both areas showed structural injury here. Cold pressor responses in CCHS subjects show extensive regions of differences from controls in areas dorsal to the hippocampus (although the spatial resolution is such that the changes could overlap the structure), from the mid-to-posterior regions on the left side [11]. Forced expiratory efforts, which evoke a pressor response, also highlighted differences in the left mid-to-posterior hippocampus [12]. Functional abnormalities in the anterior hippocampus, which showed the greatest structural injury, were not specifically identified, although the adjacent structure, the amygdala, showed differing responses to ventilatory stimuli (hypercapnia, hyperoxia, hypoxia) [13], [14], [26]. Taken together, the findings emphasize an overlap of areas with reduced volume and abnormal function in the mid and posterior regions, especially on the left.

### Cognition, Mood and Anxiety

The traditional roles of the hippocampus in cognition, especially memory consolidation, appear to be affected in many CCHS children. Working memory, including a component of spatial memory, an aspect served by the hippocampus, is impaired in CCHS [5]. Other characteristics of the syndrome are difficulties with spatial and/or mathematical concepts, which appear in 21% of a large sample of CCHS children, who also show learning difficulties at school [4]. Mood disruption, anxiety and other psychological problems are common in CCHS, and these characteristics have been related to injury and altered function of the hippocampus. In particular, the posterior hippocampus and fibers of the fornix are affected in depression [33], [34], [35], which is consistent with the posterior injury found here and the elevated level of depressive symptoms in some CCHS subjects [4], [5]. While we did not collect cognitive or mood measures, it is possible that CCHS subjects with exacerbated psychological impairments exhibit greater hippocampal deficits.

### Breathing Drive

The defining characteristics of CCHS are impaired respiratory drive during sleep and reduced or absent ventilatory responses to CO_2_; hippocampal injury may play roles in both those deficits. The drive to breathe is typically maintained in CCHS during wakefulness, although hypoventilation is present in severe cases or special circumstances, e.g., fever. Processes underlying wakefulness influences on respiration partially derive from descending forebrain sites which modify brainstem respiratory patterning; temperature influences from the hypothalamus are commonly cited, but other limbic drives participate. Affective influences, such as the perception of breathlessness, comprise one such powerful drive. The perception of dyspnea, which CCHS patients often lack, is mediated through the insular cortex, and the hippocampus plays a role [36], [37]. The hippocampus participates in normal responses to chemosensory stimuli (CO_2_ and O_2_ ventilatory challenges [13], [14], [26]), although the role of that participation is unclear.

### Autonomic Regulation

A principal characteristic of CCHS, in addition to the respiratory deficiencies, is impaired development of the autonomic nervous system; the primary targets of PHOX2B mutations are brainstem autonomic ganglia, in addition to respiratory-related neurons near the retrofacial nucleus [38], [47]. The resulting impaired development of autonomic nuclei affects both parasympathetic and sympathetic control, and leads to symptoms such as profuse sweating, low heart rate variability, syncopal episodes, impaired pupillary regulation, and poor temperature control [6], [39], [40]. The hippocampus reacts markedly to blood pressure challenges, and shows abnormal responses in CCHS patients to such challenges, although the role of the structure in blood pressure modulation is unclear [11], [12]. The structural changes found here may contribute to the altered autonomic control in the syndrome, possibly through the extensive hippocampal influences on the hypothalamus [41], [42], [43].

### Cerebrovascular Regulation and Lateralization

The lateralized hippocampal changes, with greater volume reductions on the left side, may reflect injury related to asymmetric declines in perfusion. Lateralization of overall vascular supply appears in normal subjects, and is associated with an apparent greater susceptibility for consequences of cerebrovascular disease on the left side [44]. We have evidence that cerebrovascular regulation is impaired in CCHS, since global blood oxygen level dependent signals, reflecting a combination of cerebral blood flow and oxygenation, are grossly distorted in response to CO_2_ and O_2_ challenges [45]. The nature of such dysregulation is unclear, but the consequences of altered control on vessels can be observed by eye on the MRI; the basilar artery, for example is grossly enlarged in these CCHS children, whereas other major vessels are not [46]. Faulty cerebrovascular regulation may combine with normal differentially-lateralized perfusion to elicit the preferential left-sided injury here.

### PHOX2B Mutations

The mechanisms leading to tissue loss may involve a combination of syndrome-related injury and developmental abnormalities from PHOX2B mutations. The mouse equivalent Phox2b transcription factor is expressed in the pyramidal and granule cell layers of the hippocampus [19], as well as the well-described targets in visceral ganglia and brainstem [38], [47]. In addition to genetically-related impaired growth, injury could arise during development from vascular consequences of PHOX2B actions on autonomic ganglia. However, apnea and hypoventilation in the syndrome during sleep also can exert substantial injury. Cerebellar Purkinje cells are especially sensitive to excitotoxic injury following intermittent-hypoxia exposure, with damage appearing after just 5 hours of intermittent hypoxia [48]; the process likely involves excessive excitation of climbing fibers from the inferior olive. A similar pattern may be operating in CA1 neurons from extreme excitation of Schaffer collaterals to hypoxia, in a fashion comparable to the processes which occur during seizure discharge [49]. The contributions from injury and developmental processes could not be distinguished in the present study, but previous measures of tissue integrity suggest that at least part of the changes are due to hypoxic damage [15], [16].

### Limitations

Limitations of this study include variability in outlining the hippocampus, which is common to all morphometric approaches based on manual tracing. However, our protocol differed from other approaches in that tracing was performed on sagittal views in addition to the conventional coronal aspect. The sagittal view is especially helpful in defining rostral portions of the hippocampus, which is typically unclear in coronal views. A further caution is that three of the CCHS subjects were scanned on a different scanner; no gross differences in brain images were noted between scanners. The three subjects subsequently received cardiac pacemakers, precluding further scans. The technique is also limited in accuracy of localizing volume changes to specific subfields of the hippocampus, since the surface-based differences represent volume changes below the surface, which in most areas include several subfields. Furthermore, volume reductions do not account for injury not associated with volume changes (e.g., fluid-filled lesions). Finally, the age range of the subjects covers a period of significant brain development; although careful attention was paid to age-matching, the course of hippocampal development likely added variability to the measures. However, confidence in the hippocampal findings is reinforced by the consistency with neural deficits shown by other whole-brain structural, functional, and morphometric studies.

### Summary

The hippocampus showed regional structural volume reduction in CCHS subjects, with the principal changes occurring in the rostral portion of the left hippocampus. Other bilateral alterations appeared in caudal areas of both hippocampi near the fimbria, and mid and mid-posterior regions of CA1, especially on the left. These volume changes may reflect injury from hypoxic exposure, inadequate perfusion due to autonomic dysregulation, or developmental deficits resulting from PHOX2B mutations. The consequences of the structural alterations are likely reflected in functional deficits found in the hippocampus in response to respiratory and autonomic challenges, and in psychological and autonomic impairments accompanying the syndrome.
